# Supplementary material for: Genetic structure of a germplasm for hybrid breeding in rye (Secale cereale L.)
Source: PLoS One. 2020 Oct 9;15(10):e0239541. doi: 10.1371/journal.pone.0239541 (PMC7546470; doi:10.1371/journal.pone.0239541)
Supplement: S1 Table — (DOCX) [file pone.0239541.s001.docx]

| **Line ID** | **Opposing ancestry** | **H_o_** |
| --- | --- | --- |
| R10 | 0.515 | 0.563 |
| R115 |  | 0.463 |
| R126 | 0.398 | 0.023 |
| R127 | 0.419 | 0.004 |
| R310 | 0.730 | 0.046 |
| R311 | 0.497 | 0.015 |
| R342 | 0.565 | 0.353 |
| R343 | 0.588 | 0.358 |
| NRG56 | 0.896 | 0.056 |
| NRG79 | 0.410 | 0.048 |
| NRG129 | 0.537 | 0.076 |

**S1 Table.** Opposing population ancestry from an admixture model of inferred ancestry at K set to 2 and observed residual heterozygosity (H_o_) of 11 discarded Nordic Seed hybrid rye elite breeding lines belonging to the restorer (R) and non-restorer germplasm (NRG) population.
