# Supplementary figures and images for: Genetic structure of a germplasm for hybrid breeding in rye (Secale cereale L.)
Source: PLoS One. 2020 Oct 9;15(10):e0239541. doi: 10.1371/journal.pone.0239541 (PMC7546470; doi:10.1371/journal.pone.0239541)

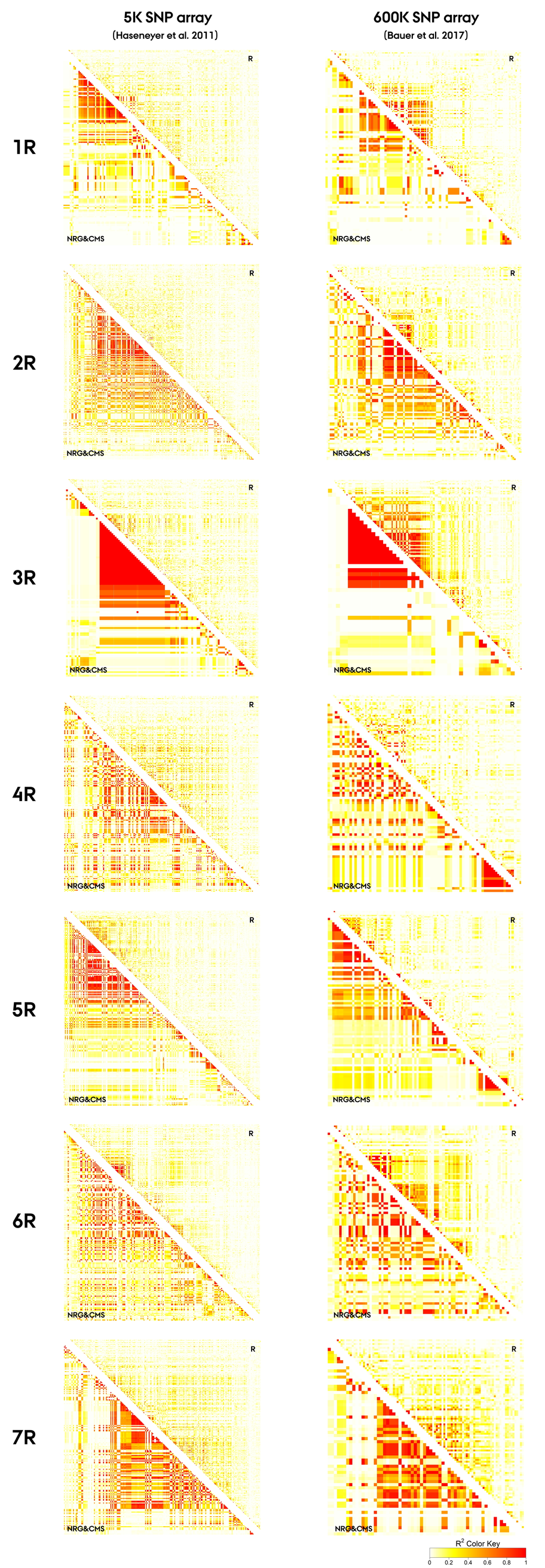

Supplement: S1 Fig — (TIF) [file pone.0239541.s002.tif]
