# Supplementary material for: Genetic structure of a germplasm for hybrid breeding in rye (Secale cereale L.)
Source: PLoS One. 2020 Oct 9;15(10):e0239541. doi: 10.1371/journal.pone.0239541 (PMC7546470; doi:10.1371/journal.pone.0239541)
Supplement: Attachment — Submitted filename: Response to Reviewers.docx [file pone.0239541.s004.docx]

**Response to Reviewers**

Dear editors and reviewers,

We greatly appreciate the editor and reviewers’ careful work and valuable comments. The comments have been very helpful for the improvement of our manuscript. The manuscript has been thoroughly revised in accordance to the comments made, and within the word document each of the modifications has been highlighted with a code to the change made, as can be seen in detail below.

Sincerely,

The authors

**Editorial reviewer**:

1. Manuscript formatting

Revision made: Manuscript has been thoroughly corrected for formatting errors according to the PLOS ONE guidelines, including file naming as mentioned

2. Financial disclosure statement

Revision made: Statement has been altered accordingly and can be found in the submitted “Cover letter” document as proscribed by PLOS One. Thank you for the elaborate instruction.

3. Competing interests statement

Revision made: Statement has been altered accordingly and can be found in the submitted “Cover letter” document as proscribed by PLOS One. Thank you for the elaborate instruction.

**Reviewer 1:**

1. Sentence at l. 15-17, l. 77-79, and, l.339-342 have been rephrased according to the suggestion provided. altered the sentence from “The first comprehensive SNP-based population study” to “The first comprehensive SNP-based population study of an elite germplasm using a fertilization control system for hybrid breeding, that is genetically different to the predominating P-type”.

Revision made: Phrasing has been altered as suggested, thank you for the observation. Our argument for the initial statement were that while Bauer *et al*. conducted a population study analysis for verification of the 600K high density array, it was more a evaluation of chip performance rather than an actual thorough population study.

2. Attach .CSV repository with marker data files

Revision made: A .CSV repository has been added with three files, sufficient to conduct all analysis reported in this study. This includes the requested information for studying the divergence in population-wise polymorphic SNPs when filtering on the raw genotype file (1).

1: geno_raw_376	Raw genotype data for all 376 lines

2: geno_376	QC-filtered genotype data for all 376 lines

3: marker_data_376	Marker position and chip-origin

3. Rephrase sentence at l. 368-369 regarding genetic distance as predictor of hybrid performance

Revision made: New sentence added incorporating the suggested transcriptome-based prediction by Frisch *et al*. as a more accurate predictor of hybrid performance. Thank you for your input, and for pointing us towards the article by Frisch *et al.* Our argument for including this sentence is to highlight that while genetic distance is not the definitively best predictor of hybrid performance, it can be readily be determined from simple SNP genotype data and applied.

4. Incomplete sentence at l. 170-171. Modified from “None of the wheat-set markers were mapped”

Revision made: Sentence completed, thank you for the keen observation.

5. Rephrase from “mother” and “father” to “parent” at l. 261, 371-372

Revision made: Phrasing has been altered as suggested, thank you for your observation.

6. Inadequate link to preceding paragraph at l. 459-462

Revision made: Sentence has been kept with minor changes, adding a “[…], however, […]” in the early onset. Let us briefly present the argument behind our decision. Shortly before the sentence in question, l. 452-455 states the following “While functional analysis in rye is impeded by poor or insufficient gene annotation […]”, hence the sentence in question concludes the paragraph by referring to the very recent reference genome by Rabanus-Wallace *et al.* and their work on *de novo* annotating the full complement of identified gene-models in rye. In near-future, information on functional annotation of the 5K-set and 600K-set markers will, therefore, be much more complete than it is at present, limited to major traits such as restoration of male-fertility. Hope that you can follow our line of thought.

7. Update literature at l. 494-498

Revision made: Sentence has been added to incorporate the mentioned study by Falke *et al*., thank you for pointing us towards the study. Believe that it has contributed towards a more thorough summary of the present knowledge of utilizing landraces and exotic material for the genetic improvement of cultivated rye.

**Reviewer 2:**

1. Alter title from ”Dissecting the Genetics of a Hybrid Rye Breeding Germplasm” to “Genetic Structure of a Germplasm for Hybrid Breeding in Rye”

Revision made: Title has been altered as suggested

2. L. 33-38, “This section could be removed”

Revision made: Section has been kept, with the argument that it provides fundamental insight in rye biology and origin which is relevant to a broader audience not intimately familiar with rye. It can be expected that this fits the description of some of PLOS Ones’ readers. Hope that you can follow our line of thought.

3. L. 72-74 (Initial manuscript submitted) or l. 65 […] in new, “This section could be removed”

Revision made: Section has been removed as suggested.

4. “Figure 7a and 7b could be removed”

Revision made: Figures kept, with the argument that they provide a visual insight in the LD structure in the 5K-set markers and 600K-set markers respectively. Hope that you can follow our line of thought.

5. Format references

Revision made: Thorough formatting has been conducted, sorry for the inconsistence!

6. Correct citation format, “*et al*” to “*et al.*”

Revision made: Formatting mistake has been corrected, again, sorry for the basic mistake.

7. Correct citation format, l. 357, l. 547-548, e.g. “Bauer, Schmutzer [1]“ to “Bauer *et al*. [1]“

Revision made: Citation format has been altered as suggested

8. Species names in italics

Revision made: Formatting mistake has been corrected

9. Correct l. 90, “Schläger” to “Schlägler”

Revision made: Misspelling has been corrected, thank you for the keen observation.
