# Supplementary material for: Genetic structure of a germplasm for hybrid breeding in rye (Secale cereale L.)
Source: PLoS One. 2020 Oct 9;15(10):e0239541. doi: 10.1371/journal.pone.0239541 (PMC7546470; doi:10.1371/journal.pone.0239541)
Supplement: Attachment — Submitted filename: Response to Reviewers.docx [file pone.0239541.s005.docx]

**Response to Reviewers**

Dear editors and reviewers,

We greatly appreciate the editor and reviewers’ careful work and valuable comments. The comments have been very helpful for the improvement of our manuscript. We have carried out a new analysis for the number of population-specific high-quality markers as requested. A minor calculation error was identified internally leading to a new number of population-specific high-quality markers as stated in the Results section line 158-159. The corrected sentence has been highlighted within the marked manuscript.

Sincerely,

The authors

**Editorial reviewer**:

**Reviewer 2:**

We have complied with all of the notions concerning data availability and hope the updated csv repository meets the reviewers expectations. In summary we have added the original marker identity, the SNP array origin (with the precise source cited in the materials and method section), chromosomal position, and interchromosomal position. Furthermore, we have added two additional csv files containing the requested population-specific high-quality markers obtained when filtering for the quality parameters on the raw geno for each of the populations respectively.
